# Functional implications of peroxisomal nitric oxide (NO) in plants

**DOI:** 10.3389/fpls.2014.00097

**Published:** 2014-03-17

**Authors:** Francisco J. Corpas, Juan B. Barroso

**Affiliations:** ^1^Departamento de Bioquímica, Biología Celular y Molecular de Plantas, Estación Experimental del Zaidín, Consejo Superior de Investigaciones CientíficasGranada, Spain; ^2^Departamento de Bioquímica y Biología Molecular, Facultad de Ciencias Experimentales, Universidad de JaénJaén, Spain

**Keywords:** nitric oxide, nitrosative stress, nitrosoglutathione, oxidative stress, peroxynitrite, peroxisomes, reactive nitrogen species

Nitric oxide (NO), peroxynitrite (ONOO^−^), and *S*-nitrosoglutathione (GSNO) are components of a family of molecules which have important signaling functions in higher plants under physiological and stress conditions because directly or indirectly can mediate post-translational modifications including binding to metal centers, *S*-nitrosylation of thiol groups and nitration of tyrosine (Lamattina et al., 2003; Besson-Bard et al., 2008; Baudouin, 2011).

During the last 10 years or so, different sets of data indicate surprising new findings in relation to the enzymatic composition and functions of plant peroxisomes. One of these discoveries was the presence of an L-arginine-dependent NO synthase activity which initially shows that these organelles house a complete NO metabolism that participates in the physiology of whole plants under normal and adverse environmental conditions (Barroso et al., 1999; Corpas et al., 2004).

## Peroxisomal nitric oxide participates in a whole array of physiological processes

**Figure 1:**
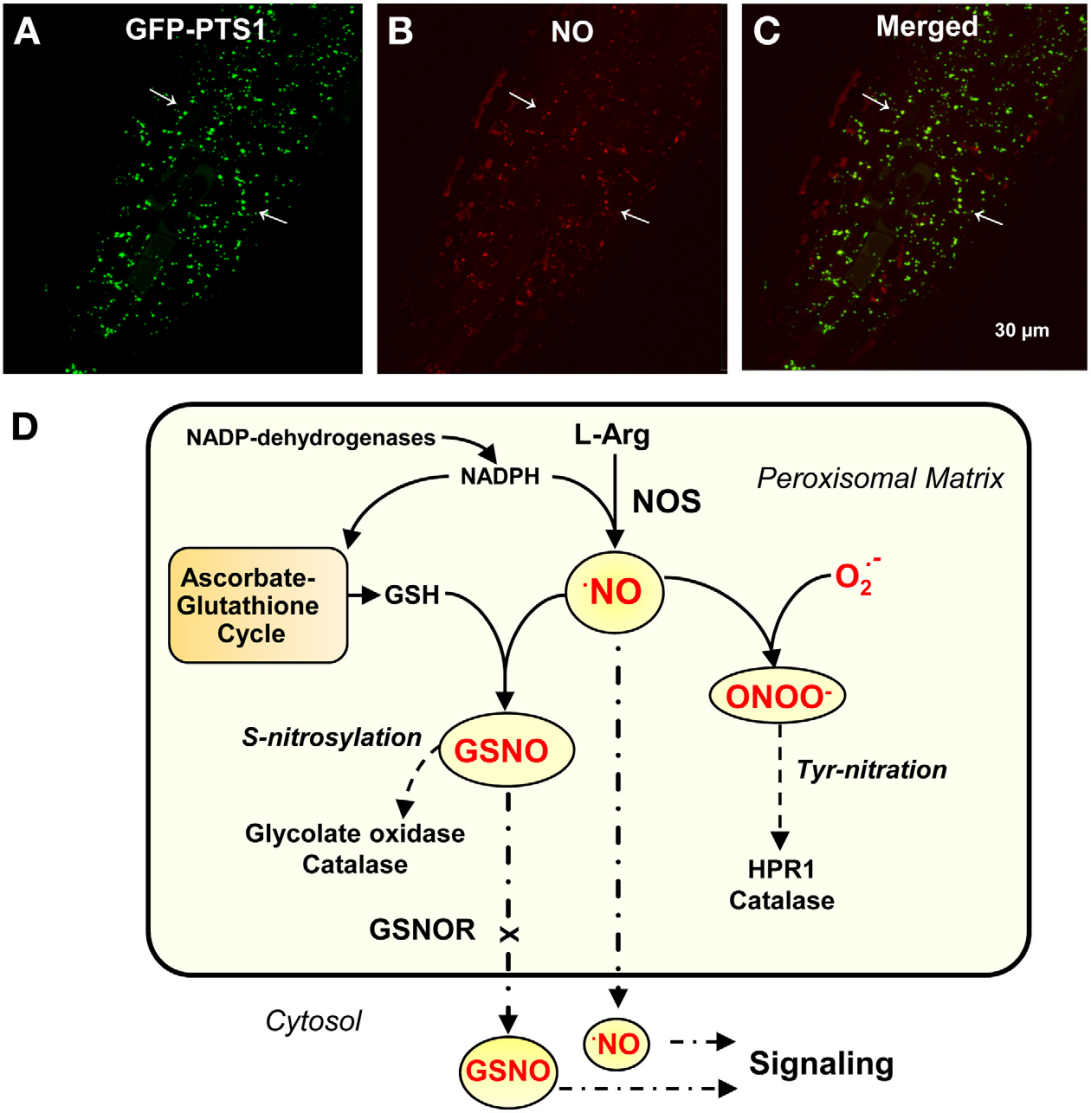
**Peroxisomal localization by confocal laser scanning microscope (CLSM) of molecules involved in NO metabolism**. **(A)** CLSM *in vivo* detection of peroxisomes (green color) in primary roots of Arabidopsis seedlings expressing GFP-PTS1 (green fluorescent protein fused with peroxisomal targeting signal 1). **(B)** CLSM *in vivo* detection of NO (red color) with DAR-AM AM in the field showed in panel **(A)**. **(C)** merged images of panels **(A,B)**. Arrows indicate representative punctate spots corresponding to NO and peroxisome localization. **(D)** Model proposed for the metabolism and signaling function of nitric oxide (NO) and *S*-nitrosoglutathione (GSNO) in plant peroxisomes. Reproduced, with permission, from *Plant Physiol*. 151:2083–2094 (Copyright American Society of Plant Biologists) for panels **(A–C)**.

Larger than a mitochondrion and smaller that a chloroplast, plant peroxisomes observed under an electron microscope are characterized as having a very simple structure made up of a single membrane that includes a granular matrix and sometimes a crystal structure (Tenberge and Eising, 1995; Usuda et al., 1999). However, from a metabolic perspective, peroxisomes possess an important and complex enzymatic composition characterized by plasticity which is adaptable to the plant organ, development stage, and/or environmental conditions (Fulda et al., 2004; Ma et al., 2006; León, 2008; Babujee et al., 2010; Hu et al., 2012). However, with the unexpected identification of peroxisomal proteins, new functions for these organelles have been proposed (Nowak et al., 2004; Reumann et al., 2007; Sørhagen et al., 2013). Given peroxisomal L-arginine-nitric oxide synthase (NOS) activity has the same cofactor requirements as animal NOS responsible for the endogenous generation of NO (Figures 1A–C) (Corpas et al., 2009 and references therein), it has been suggested that these organelles are a source of NO which can regulate peroxisomal metabolism and also be a source of long-distance signal molecules that participate in cross-talk between the different subcellular compartments (del Río, 2011). In addition, data from different areas of plant research suggest that peroxisomal NO participates in an array of physiological functions and in different organs such as leaf senescence, pollen tube growth (Prado et al., 2004), auxin-induced root organogenesis (Schlicht et al., 2013) as well as being involved in the mechanism of response to abiotic stress conditions such as salinity and cadmium stress (Corpas et al., 2009; Corpas and Barroso, 2014).

## Peroxisomal nitric oxide metabolism

Recently, cellular and biochemical approaches have shown the presence of new components involved in the peroxisomal metabolism of NO such as ONOO^−^, *S*-nitrosoglutathione (GSNO), GSNO reductase and protein nitration (Heijnen et al., 2006; Barroso et al., 2013; Corpas and Barroso, 2014) which contribute to a more complete picture of the peroxisomal metabolism. It has also been demonstrated that Arabidopsis plants under cadmium stress, peroxisomal peroxynitrite, NO, and superoxide anion (O^·−^_2_) are overproduced, suggesting that peroxisomes participate in the nitro-oxidative stress response to this heavy mental (Corpas and Barroso, 2014). Given that NO and related molecules can mediate post-translational modifications, mainly nitration and *S*-nitrosylation, several proteomic studies of different plant species have also shown that some peroxisomal proteins are potential targets of post-translation modifications mediated by NO-derived molecules. Thus, a proteomic analysis of isolated pea leaf peroxisomes has shown that the NADH-dependent hydroxypyruvate reductase (HPR1) involved in the photorespiration pathway is negatively modulated by tyrosine nitration, specifically Tyr198, which affects the binding of the coenzyme (Corpas et al., 2013). This suggests a clear connection between NO metabolism and photorespiration. *In vitro* assays using NO donors also revealed that several peroxisomal enzymes, including catalase, malate dehydrogenase, glycolate oxidase, and hydroxypyruvate reductase, are also potential candidates of *S*-nitrosylation (Ortega-Galisteo et al., 2012).

## Concluding remarks and future research

Accumulated data obtained during the last 10 years or so confirm that plant peroxisomes possess a whole metabolic mechanism related to NO metabolism which complements our knowledge of the reactive oxygen species (ROS) metabolism. Therefore, our understanding of the biological chemistry of NO in peroxisomes presented in Figure 1D will now provide a framework to comprehend how these NO-derived molecules participate in the plant development process (Prado et al., 2004; Schlicht et al., 2013) and to understand the mechanism of response to environmental stress (Corpas and Barroso, 2014). Further research is required to elucidate the nature of finely-tuned endogenous regulation of peroxisomal components.
